# Effects of Donepezil on the Musculoskeletal System in Female Rats

**DOI:** 10.3390/ijms24108991

**Published:** 2023-05-19

**Authors:** Piotr Londzin, Marcin Trawczyński, Urszula Cegieła, Zenon P. Czuba, Joanna Folwarczna

**Affiliations:** 1 Department of Pharmacology, Faculty of Pharmaceutical Sciences in Sosnowiec, Medical University of Silesia, Katowice, Jagiellońska 4, 41-200 Sosnowiec, Poland; marcinpas90@gmail.com (M.T.); ucegiela@sum.edu.pl (U.C.); jfolwarczna@sum.edu.pl (J.F.); 2 Department of Microbiology and Immunology, Faculty of Medical Sciences in Zabrze, Medical University of Silesia, Katowice, Jordana 19, 41-808 Zabrze, Poland; zczuba@sum.edu.pl

**Keywords:** donepezil, musculoskeletal system, estrogen deficiency, osteoporosis, rats

## Abstract

The extension of human life makes it more and more important to prevent and treat diseases of the elderly, including Alzheimer’s disease (AD) and osteoporosis. Little is known about the effects of drugs used in the treatment of AD on the musculoskeletal system. The aim of the present study was to investigate the effects of donepezil, an acetylcholinesterase inhibitor, on the musculoskeletal system in rats with normal and reduced estrogen levels. The study was carried out on four groups of mature female rats: non-ovariectomized (NOVX) control rats, NOVX rats treated with donepezil, ovariectomized (OVX) control rats and OVX rats treated with donepezil. Donepezil (1 mg/kg p.o.) was administered for four weeks, starting one week after the ovariectomy. The serum concentrations of CTX-I, osteocalcin and other biochemical parameters, bone mass, density, mineralization, histomorphometric parameters and mechanical properties, and skeletal muscle mass and strength were examined. Estrogen deficiency increased bone resorption and formation and worsened cancellous bone mechanical properties and histomorphometric parameters. In NOVX rats, donepezil decreased bone volume to tissue volume ratio in the distal femoral metaphysis, increased the serum phosphorus concentration and tended to decrease skeletal muscle strength. No significant bone effects of donepezil were observed in OVX rats. The results of the present study indicate slightly unfavorable effects of donepezil on the musculoskeletal system in rats with normal estrogen levels.

## 1. Introduction

The most recent data indicate that there are over 58 million people worldwide living with Alzheimer’s disease (AD) or other dementias, and this number is expected to reach at least 152 million by 2050 [1]. AD is a progressive, irreversible neurodegenerative disorder clinically characterized by memory loss, cognitive impairment, executive dysfunction, behavioral changes and personality disorders [2]. The main pathophysiological processes in AD include an aberrant accumulation of extracellular β-amyloid (Aβ) protein in senile plaques, followed by the formation of intracellular neurofibrillary tangles consisting of abnormally hyperphosphorylated tau protein [2,3,4,5,6,7]. These processes are accompanied by inflammation and synaptic and mitochondrial disorders leading to, among other things, cognitive impairment due to cholinergic, adrenergic and glutamatergic transmission dysfunction [8].

Osteoporosis is the most common metabolic disorder in humans, characterized by a decrease in bone mass and strength and a disruption of bone microarchitecture, resulting from disorders of the bone remodeling process [9,10], which is regulated by an endocrine, paracrine and autocrine signaling and autonomic nervous system (adrenergic and cholinergic branches) [11]. Consequently, increased susceptibility to fractures and a significant deterioration in the quality of life are observed in patients [10]. The most common type of osteoporosis is postmenopausal osteoporosis, which develops as a result of estrogen deficiency in women [12].

There are numerous links between AD and osteoporosis [13,14,15,16,17,18,19,20]. The prevalence of both AD and osteoporosis is higher in women than in men [13]. Both diseases relate to aging [18] and result in a significant morbidity and mortality rate [21]. There are epidemiological reports indicating an association between low bone mineral density (BMD) or osteoporosis and AD [13,14,20]. There is also an interplay between musculoskeletal deficits and cognitive impairment (observed in AD), as has been recently comprehensively reviewed [15]. A meta-analysis of cohort studies indicated that there is also strong evidence that AD is a risk factor for hip fracture [22]. In addition, osteoporosis is associated with a higher risk of cognitive impairment/AD [13,19]. Possible connections between osteoporosis and AD include disorders of cholinergic transmission in the central nervous system (CNS) and the skeletal system. Dysfunctions in cholinergic neurotransmission contribute to the learning, memory and attention impairment observed in AD [23]. Although the role of the sympathetic nervous system in the regulation of the skeletal system metabolism is better understood, activation of the parasympathetic (or non-neuronal cholinergic) system is thought to be associated with beneficial effects on bone mass accrual [11,24,25,26,27].

Although there are many causative drugs under investigation in different stages of clinical trials [6,7,28,29], mainly symptomatic drugs are used. These are acetylcholinesterase inhibitors (AchEIs)—donepezil, galantamine and rivastigmine and a non-competitive N-methyl-D-aspartate receptor antagonist—memantine [7,28,30,31]. Little is known about the effect of drugs used in the treatment of AD on the skeletal system.

Previous studies on the effects of cholinergic signaling on bone suggested its positive effects; for comprehensive reviews see [25,26]. Limited data concerning the use of cholinergic drugs (including donepezil) in patients with AD indicate reduced fracture risk and improved bone healing [32,33,34,35,36]. However, the effect on bone may have resulted from amelioration of the AD-related disorders (memory loss, cognitive impairment and others) in patients. Experimental studies performed so far to evaluate the effects of AchEI on bone led to inconsistent results [37,38,39]. Although favorable effects of prolonged treatment with donepezil were demonstrated in healthy young female mice [38], donepezil hindered bone healing in female rats [37].

Taking into account that the majority of AD female patients are postmenopausal and estrogen deficiency may contribute to brain aging and the development of AD [40], it seems important to evaluate the effects of drugs used in the treatment of AD in conditions of estrogen deficiency. Therefore, the aim of the present study was to investigate the effect of donepezil, as a representative of AchEIs, on the skeletal system in healthy adult female rats with normal and reduced estrogen levels (a model of postmenopausal osteoporosis). Considering the close interactions between bone and skeletal muscle [41,42], the study also involved the effects of donepezil on the skeletal muscle mass and strength in rats.

## 2. Results

### 2.1. Effects of Donepezil on the Body Mass Gain, Mass of Internal Organs and Serum General Biochemical Parameters in NOVX and OVX Rats

Estrogen deficiency increased the body mass gain after four weeks of drug or vehicle administration in the OVX control rats in comparison to the NOVX control rats (Table 1). Oral administration of donepezil for four weeks at a dose of 1 mg/kg did not affect the body mass gain in NOVX and OVX rats in relation to the appropriate control rats.

In estrogen-deficient rats, a decreased mass of the uterus and increased mass of the thymus were noted compared to the NOVX control rats. There was no significant effect of estrogen deficiency on the mass of the liver in relation to the NOVX controls. Additionally, no significant effects of donepezil treatment were demonstrated on the mass of the uterus, thymus or liver in neither NOVX or OVX rats.

Estrogen deficiency increased the serum total cholesterol level and decreased the uric acid concentration in the OVX control rats compared to the NOVX control rats (Table 2). Estrogen deficiency also tended to decrease the lactate dehydrogenase (LDH) activity in the OVX controls in relation to the NOVX controls. In OVX rats, decreased activity of alanine aminotransferase (ALT) and decreased concentrations of total protein and urea in the serum compared to the NOVX controls were noted (significant main effects of OVX in the two-way ANOVA). There was no effect of estrogen deficiency on other investigated serum biochemical parameters.

The serum LDH activity tended to decrease in the donepezil-treated NOVX rats in relation to the NOVX control rats.

In the OVX rats treated with donepezil, the LDH activity increased in comparison to the OVX controls. There was also a tendency to decrease the glucose level in the donepezil-treated OVX rats compared to the OVX control rats. No effects of donepezil treatment were demonstrated on other investigated serum biochemical parameters in neither NOVX or OVX rats.

### 2.2. Effects of Donepezil on the Serum Bone Metabolic Parameters in NOVX and OVX Rats

Estrogen deficiency increased the serum levels of bone formation markers (osteocalcin and alkaline phosphatase (ALP)) and the serum level of bone resorption markers (C-terminal telopeptide fragments of type I collagen—CTX-I) in OVX rats in relation to the NOVX control rats (Table 3). Increased serum phosphorus concentration was noted in OVX rats compared to the NOVX controls.

The serum phosphorus concentration increased in the NOVX rats treated with donepezil compared to the NOVX controls. No significant effects on other investigated serum bone metabolic parameters were observed in the donepezil-treated NOVX or OVX rats.

### 2.3. Effects of Donepezil on Mechanical Properties of the Proximal Tibial Metaphysis in NOVX and OVX Rats

**Figure 1:**
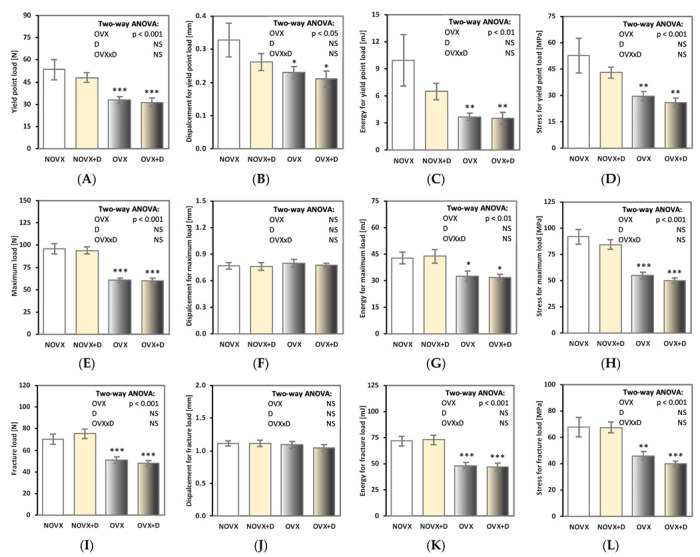
Effects of donepezil (1 mg/kg p.o.) administered for 4 weeks on mechanical properties of the proximal tibial metaphysis in non-ovariectomized and ovariectomized rats. (**A**–**D**) Yield point load, displacement, energy and stress for the yield point. (**E**–**H**) Maximum load, displacement, energy and stress for the maximum load. (**I**–**L**) Fracture load, displacement, energy and stress for the fracture load. The results are presented as means ± standard error of the mean (SEM). NOVX—non-ovariectomized control rats; NOVX + D—non-ovariectomized rats treated with donepezil; OVX—ovariectomized control rats; OVX + D—ovariectomized rats treated with donepezil. Two-way analysis of variance (ANOVA) followed by Fisher’s LSD test were used for evaluation of the significance of the results. NS—non-significant in the two-way ANOVA. * *p* < 0.05, ** *p* < 0.01, *** *p* < 0.001—in comparison to the NOVX control rats (NOVX group).

**Figure 2:**
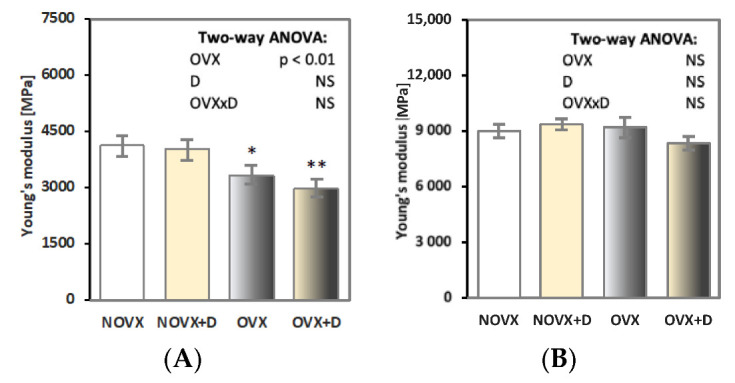
Effects of donepezil (1 mg/kg p.o.) administered for 4 weeks on Young’s modulus in the proximal tibial metaphysis (**A**) and femoral diaphysis (**B**) in non-ovariectomized and ovariectomized rats. The results are presented as means ± standard error of the mean (SEM). NOVX—non-ovariectomized control rats; NOVX + D—non-ovariectomized rats treated with donepezil; OVX—ovariectomized control rats; OVX + D—ovariectomized rats treated with donepezil. Two-way analysis of variance (ANOVA) followed by Fisher’s LSD test were used for evaluation of the significance of the results. NS—non-significant in the two-way ANOVA. * *p* < 0.05, ** *p* < 0.01—in comparison to the NOVX control rats (NOVX group).

Estrogen deficiency worsened the mechanical properties of the proximal tibial metaphysis (built mostly of cancellous bone; Figure 1A–L and Figure 2A). Decreases in the values of the load, displacement, energy and stress for the yield point and the load, energy and stress for the maximum load and fracture points in the OVX controls in relation to the NOVX controls were demonstrated. Moreover, the value of Young’s modulus decreased in estrogen-deficient rats (Figure 2A).

Donepezil administration did not affect the mechanical properties of the proximal tibial metaphysis in neither NOVX or OVX rats.

### 2.4. Effects of Donepezil on Mechanical Properties of the Femoral Diaphysis and Femoral Neck in NOVX and OVX Rats

The unfavorable effects of estrogen deficiency on mechanical properties were not observed in compact bone of the femoral diaphysis or in the femoral neck (built of compact and cancellous bone) (Table 4 and Figure 2B). There were also no significant effects of donepezil administration on the mechanical properties of the femoral diaphysis or femoral neck in neither NOVX or OVX rats.

### 2.5. Effects of Donepezil on the Length, Mass, Composition, Mineralization and Density of Bones in NOVX and OVX Rats

In the OVX control rats, decreases in the bone mineral mass/bone mass ratio, bone density and bone mineral density in the femur in relation to the NOVX control rats were demonstrated (Table 5). Bone density and bone mineral density in the tibia decreased in the OVX control rats compared to the NOVX control rats (Supplementary Table S1). Moreover, bone mineral mass, bone density and bone mineral density in the vertebra decreased (Supplementary Table S2) and the length of the tibia increased in OVX rats in relation to NOVX rats (significant main effects of OVX in the two-way ANOVA). In addition, decreased bone mineral mass and increased mass of bone water/bone mass ratio in the femur were noted in estrogen-deficient rats compared to NOVX rats (significant main effects of OVX in the two-way ANOVA). Estrogen deficiency did not affect other investigated parameters in the femur, tibia or vertebra in the OVX controls in relation to the NOVX controls.

Donepezil treatment decreased calcium content in the bone mineral of the vertebra in NOVX rats in relation to the NOVX controls. The length of the tibia tended to decrease in the donepezil-treated NOVX rats in comparison to the NOVX controls. Donepezil administration did not affect other parameters concerning bone mass, density and mineralization in the femur, tibia or vertebra in NOVX rats.

Donepezil administration to OVX rats increased the phosphorus content in the bone mineral of the femur compared to the OVX control rats. Phosphorus content in the bone mineral of the tibia and vertebra was not affected by donepezil treatment in OVX rats. In addition, the calcium and magnesium content in the bone mineral of the femur, tibia and vertebra were not changed in the donepezil-treated OVX rats compared to the OVX controls. Donepezil administration to OVX rats did not affect other investigated parameters in the femur, tibia or vertebra compared to the OVX controls.

### 2.6. Effects of Donepezil on the Histomorphometric Parameters of Compact Bone in NOVX and OVX Rats

Estrogen deficiency increased the transverse cross-sectional area of the marrow cavity/total diaphysis area ratio (Ma.Ar/Tt.Ar) in the femoral diaphysis in the OVX controls compared to the NOVX controls (Table 6). Estrogen deficiency also increased the transverse cross-sectional area of the marrow cavity (Ma.Ar) and periosteal MAR (Ps.MAR) in the femoral diaphysis and increased the transverse cross-sectional area of the cortical bone (Ct.Ar), Ma.Ar and the transverse cross-sectional area of the total area (Tt.Ar) in the tibial diaphysis in relation to NOVX rats (Supplementary Table S3; significant main effects of OVX in the two-way ANOVA).

No effects of donepezil administration on the histomorphometric parameters of compact bone (femoral and tibial diaphysis) were observed in neither NOVX or OVX rats in relation to the appropriate controls.

### 2.7. Effects of Donepezil on the Histomorphometric Parameters of Cancellous Bone and Epiphyseal Cartilage in NOVX and OVX Rats

**Figure 3:**
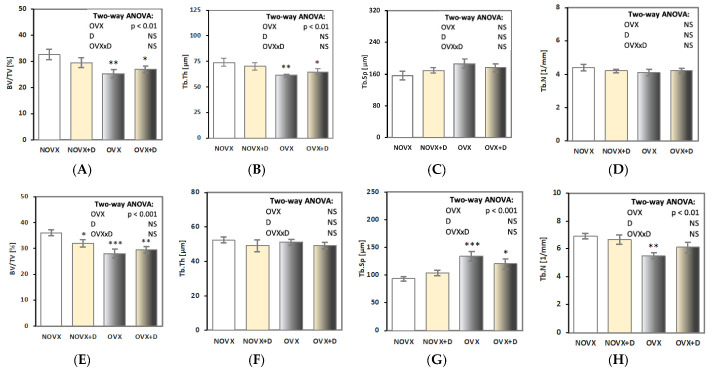
Effects of donepezil (1 mg/kg p.o.) administered for 4 weeks on histomorphometric parameters of cancellous bone in the femoral epiphysis (**A**–**D**) and femoral metaphysis (**E**–**H**) in non-ovariectomized and ovariectomized rats. The results are presented as means ± standard error of the mean (SEM). NOVX—non-ovariectomized control rats; NOVX + D—non-ovariectomized rats treated with donepezil; OVX—ovariectomized control rats; OVX + D—ovariectomized rats treated with donepezil. Two-way analysis of variance (ANOVA) followed by Fisher’s LSD test were used for evaluation of the significance of the results. NS—non-significant in the two-way ANOVA. * *p* < 0.05, ** *p* < 0.01, *** *p* < 0.001—in comparison to the NOVX control rats (NOVX group).

Strong deterioration of the microstructure of cancellous bone in the femoral epiphysis and metaphysis was demonstrated in estrogen-deficient rats (Figure 3A–H). In the femoral epiphysis, decreased bone volume/tissue volume ratio (BV/TV) and trabecular thickness (Tb.Th) were demonstrated in the OVX control rats in comparison to the NOVX control rats. In the femoral metaphysis, decreased BV/TV and trabecular number (Tb.N) and increased trabecular separation (Tb.Sp) were demonstrated.

Donepezil administration decreased BV/TV in the femoral metaphysis in NOVX rats in comparison to the NOVX control rats. There was no significant effect of donepezil treatment on the histomorphometric parameters of cancellous bone in OVX rats.

The width of the hypertrophic zone of the epiphyseal cartilage in the femur was reduced in the OVX controls in relation to the NOVX controls (Table 7). The effects of donepezil treatment were different in NOVX and OVX rats: in the donepezil-treated NOVX rats, a decrease in the hypertrophic zone width was noted, while an opposite effect was observed in the donepezil-treated OVX rats—an increase in the hypertrophic zone width, compared to appropriate control rats (normalization in relation to the NOVX controls). No effects of estrogen deficiency or donepezil treatment were observed on the width of the reserve and proliferative zones of the epiphyseal cartilage.

### 2.8. Effects of Donepezil on the Grip Strength and the Mass of Skeletal Muscle in NOVX and OVX Rats

**Figure 4:**
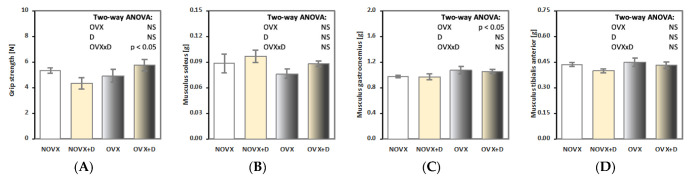
Effects of donepezil (1 mg/kg p.o.) administered for 4 weeks on the grip strength (**A**) and mass of skeletal muscles: the musculus soleus (**B**), musculus gastrocnemius (**C**) and musculus tibialis anterior (**D**) in non-ovariectomized and ovariectomized rats. The results are presented as means ± standard error of the mean (SEM). NOVX—non-ovariectomized control rats; NOVX + D—non-ovariectomized rats treated with donepezil; OVX—ovariectomized control rats; OVX + D—ovariectomized rats treated with donepezil. Two-way analysis of variance (ANOVA) followed by Fisher’s LSD test were used for evaluation of the significance of the results. NS—non-significant in the two-way ANOVA.

Estrogen deficiency tended to increase the mass of musculus gastrocnemius in the OVX control rats in comparison to the NOVX control rats, with no such effect on other skeletal muscles or the grip strength (Figure 4).

In NOVX rats, donepezil tended to decrease the grip strength in relation to the NOVX control rats (Figure 4A). No significant effects of donepezil administration on the grip strength or the mass of skeletal muscles were observed in OVX rats.

### 2.9. Effects of Donepezil on the Serum Concentrations of Cytokines in NOVX and OVX Rats

Estrogen deficiency increased the serum vascular endothelial growth factor (VEGF) level in the OVX controls compared to the NOVX controls (Table 8). In addition, a tendency to decrease the serum level of macrophage inflammatory protein-3a (MIP-3a; CC motif chemokine ligand 20 (CCL20)) in the OVX controls compared to the NOVX controls was noted. Moreover, estrogen deficiency, independently of the treatment, increased the serum level of the following cytokines: interleukin-1β (IL-1β), IL-7, granulocyte-macrophage colony-stimulating factor (GM-CSF), monocyte chemoattractant protein-1 (MCP-1; CC motif chemokine ligand 2 (CCL2))) and growth-regulated oncogene/keratinocyte chemoattractant (GRO/KC; CXC motif chemokine ligand 1 (CXCL1)) and decreased the serum interferon-γ (IFN-γ) level (significant main effects of estrogen deficiency in the two-way ANOVA).

No effect of donepezil treatment on the serum concentrations of cytokines was demonstrated in NOVX rats compared to the NOVX control rats. The serum levels of MCP-1 (CCL2) and MIP-3a (CCL20) increased in the donepezil-treated OVX rats in comparison to the OVX control rats. Moreover, tendencies to increase the serum levels of GM-CSF and tumor necrosis factor α (TNF-α) in the donepezil-treated OVX rats compared to the OVX control rats were demonstrated.

## 3. Discussion

The present study concerned the effects of donepezil, the most frequently used AchEI in AD [43], which is prescribed for the symptomatic treatment of patients with mild to severe disease [28,44], on the musculoskeletal system in female rats. It is blood-brain-barrier (BBB)-permeable AchEI [45] that reversibly inhibits acetylcholinesterase (AchE), the main enzyme that breaks down acetylcholine (Ach), increasing the concentration of Ach at the synapse [28]. Ach is a neurotransmitter in the CNS and at the synapses of the autonomic ganglia, the neuromuscular junction and the smooth muscle of visceral organs, acting on muscarinic and nicotinic cholinergic receptors [11,46].

The present study was conducted on NOVX and OVX mature female rats (15 weeks old at the start of the experiment). The model of ovariectomized, 3-month-old rats is one of the most commonly used models of changes observed in postmenopausal women [47]. Due to the fast bone metabolism in rats, the changes in the skeletal system induced by estrogen deficiency can be observed in a relatively short time (5 weeks in the present study). The intact (NOVX) rats of exactly the same age were used as the controls to the OVX model. The rat models do not fully mimic the changes taking place in the human skeletal system. Although rats of that age are sexually mature, longitudinal bone growth still takes place. Cancellous bone remodeling seems similar in rats and humans, whereas in compact bone, the Haversian remodeling is low [48].

The dose of donepezil (1 mg/kg p.o.) used in the present study was chosen based on previous experimental studies on rats, in which it exerted favorable effects on CNS function [49,50,51,52] and doses used in the treatment of AD patients [43,44,53,54,55]. Donepezil was administered to rats for four weeks. A four-week treatment in adult rats corresponds to approximately 2.5 years of the human life span [56]. This is a sufficient period to study the effects of drugs on the skeletal system in conditions of estrogen deficiency, as previously reported [57,58,59].

The present study was carried out on healthy, non-AD rats with normal and reduced estrogen levels. The healthy rats were chosen because the use of drugs for AD treatment in rats with the experimentally induced sporadic form of AD or in genetic AD models, leading to improvement in disordered CNS function parameters, may distort the potential direct effects on the skeletal system; similarly, the beneficial effects of AchEIs on AD-related disorders in patients may affect the effects of these drugs on the skeletal system (fracture rate). Moreover, donepezil is a drug that is not only used in the treatment of patients with AD and AD-related dementias but also in patients with cognitive impairment induced by cancer therapy [60], in non-AD patients with obstructive sleep apnea [61] and in the treatment of patients with vascular dementia [62,63].

The role of the autonomic nervous system in the regulation of bone metabolism has been acknowledged for many years [11,64]. However, the exact mechanisms by which the autonomic nervous system affects the skeletal system still need to be clarified. Generally, adrenergic system activation was reported to favor bone loss (reduced osteoblast proliferation and differentiation and increased osteoclastogenesis) [11,64], whereas cholinergic activity favored bone mass accrual and bone formation [25,65] and inhibited bone resorption by stimulating osteoclast apoptosis [66]. Both cholinergic receptors (nicotinic and muscarinic) were found on osteoblasts and osteoclasts [25,26]. In addition, AchE is widely expressed in non-neuronal cells such as bone cells [25,26,67,68].

The results of the present study indicate that donepezil administered at a dose of 1 mg/kg p.o. for four weeks exerted slight unfavorable effects on the skeletal system of NOVX rats. Donepezil disordered cancellous bone microarchitecture, decreasing BV/TV in the femoral metaphysis in NOVX rats. A slight decrease in the calcium content in the vertebra mineral only was noted. Additionally, in NOVX rats, a decrease in the width of the hypertrophic zone of the epiphyseal cartilage was demonstrated. Moreover, donepezil increased the serum phosphorus level in NOVX rats compared to the NOVX control rats.

The results of the present study in NOVX rats are inconsistent with the results of donepezil demonstrated in clinical studies [32,33,34,35,36], in which, however, the drug-induced decrease in fracture rate could be an effect of improvement of the disease symptoms. The results are also at variance with previous experimental studies on the donepezil effect [38,39,69] and other studies concerning changes in cholinergic transmission in the skeletal system [24,65,66,70]. So far, the bone effects of donepezil have been investigated in experimental studies in mice [38,39], in rats [37] and in vitro [39,69]. In vitro, donepezil suppressed osteoclastogenesis and reduced RANK expression in murine bone marrow macrophages [39] and increased expression of osteoblast markers and promoted osteogenic differentiation of human mesenchymal stem cells [69]. However, the effects exerted by donepezil may be more complex; not only must the direct effects of an increased Ach level on muscarinic and nicotinic receptors in bone cells be considered but also the effects of an increased Ach level on central cholinergic receptors and on nicotinic receptors in the autonomic ganglia. Increased activation of nicotinic receptors in the ganglia of the autonomic nervous system leads to a resultant of the sympathetic and parasympathetic system activation. Low nicotine concentrations stimulated the proliferation of osteoblasts through nicotinic receptors in vitro, while higher concentrations (such as those that may occur in cigarette smokers) reduced the proliferation of osteoblasts [71]. A high nicotine concentration exerted a negative effect on bone healing in rats [72]. Agonists of cholinergic receptors (muscarinic and nicotinic) reduced alkaline phosphatase (ALP) activity in osteoblasts [68]. However, ALP gene expression in bone marrow stromal cells isolated from long bones of old female mice did not change after treatment with AchEIs (galantamine and pyridostigmine) [70].

Short-term administration of donepezil (2 mg/kg i.p. for three days) reduced bone resorption (preventing RANKL-induced bone loss) in male mice [39]. Donepezil (0.6 mg/kg i.p. administered for four weeks) favored bone mass accrual and improved mechanical properties of the tibia in young healthy female mice in vivo [38]. Administration of galantamine, another BBB-permeable AchEI, improved the histomorphometric parameters of cancellous bone (increased BV/TV in the femur and vertebra), improved bone formation parameters (increased osteoblast surface and number, bone formation rate/bone surface and mineralized surface/bone surface in vertebral trabecular bone) and increased trabecular bone mass in adult female mice [70]. However, no effect of galantamine on BV/TV in the femur or vertebra in adult male mice and aging male and female mice was observed. In addition, inconsistent with the results of the present study, no effects of neither galantamine or pyridostigmine on the serum phosphorus level were noted [70]. On the other hand, pyridostigmine (BBB-impermeable AchEI, contrary to galantamine and donepezil) worsened the histomorphometric parameters of cancellous bone (decreased values of BV/TV in the femur and vertebra) and promoted bone resorption (increased the receptor activator of nuclear factor κB ligand (RANKL) to osteoprotegerin (OPG) ratio and IL-1β mRNA expression and increased osteoclast surface and number in vertebral trabecular bone) in adult female mice [70]. Consistently with the generally beneficial effects of an increase in cholinergic transmission, its decrease led to unfavorable effects. The low bone mass associated with increased bone resorption (increased number of tartrate-resistant acid phosphatase (TRAP)-positive osteoclasts and the serum CTX-I level) was demonstrated in α_2_-subunit of nicotinic-receptor-deficient male mice [66]. In vivo, the values of Tb.Th decreased in vertebral trabecular bone in female and male mice with a reduced Ach brain level (in female mice, reduced values of BV/TV in vertebral trabecular bone were also noted) [70].

Together, the previous studies suggested favorable effects of central cholinergic transmission and a possibility of unfavorable effects of peripheral cholinergic transmission in experimental animals. In addition, clinical studies demonstrated that AchEI treatment reduced fracture risk [33,34,35,36] or improved fracture healing in AD patients [32]. According to a recent review [45], the clinical and experimental studies concerning donepezil administration have shown that donepezil decreased fracture risk, which may have resulted from the association between AchE activity and osteoblast function and osteoclast differentiation.

On the other hand, the results of the present study are consistent with the results of Al-Hamed et al. [37], who demonstrated that donepezil (0.6 mg/kg/day sc.) administered for two weeks decreased implant osseointegration and bone healing in female rats. This is the only study that is consistent with the results obtained in the present study. Moreover, the results of another study concerning the effects of BBB-impermeable AchEI (pyridostigmine) on bone in mice indicate some similarities with the results of our study on donepezil (BBB-permeable AchEI), suggesting that the effect observed here may have been due to greater peripheral activity of donepezil in rats than in mice [70].

Estrogen deficiency occurring in postmenopausal women causes changes in bone remodeling leading to decreased bone mass, disordered microstructure and decreased strength [9,10,12]; moreover, some data indicate the possible effects of estrogen deficiency on cognition in humans [73,74,75]. Since most AD female patients are postmenopausal, they are at risk of postmenopausal osteoporosis [12]. The model of bilaterally OVX rats enables the study of the effects of donepezil on bones in conditions of estrogen deficiency.

As previously described, a bilateral ovariectomy increased bone resorption (increasing the serum CTX-I level) [58,59] and bone formation (increasing the osteocalcin level and ALP activity) [57] in rats. Profound osteoporotic microstructure changes were demonstrated in cancellous bone in the femoral epiphysis (decreased BV/TV and Tb.Th) and in the femoral metaphysis (decreased BV/TV and Tb.N and increased Tb.Sp). The changes led to a reduction in cancellous bone strength (the proximal tibial metaphysis). Less adverse effects of estrogen deficiency were observed in compact bone: increased Ma.Ar/Tt.Ar ratio in the femoral diaphysis, without the effect on compact bone mechanical properties (the femoral diaphysis). Decreased bone mineral density and bone density were noted in the long bones in the OVX control rats and no significant effect of estrogen deficiency was observed on bone mass, macrometric parameters or composition. Moreover, in the present study, it was demonstrated that estrogen deficiency unfavorably affected the epiphyseal cartilage leading to a decrease in the width of the hypertrophic zone. In addition, in the OVX control rats, increased serum phosphorus concentration compared to the NOVX controls was demonstrated. Additionally, other characteristic changes were noted: an increase in the body mass gain, a decrease in the mass of the uterus and an increase in the mass of the thymus in OVX rats in relation to the NOVX controls.

Contrary to NOVX rats, no unfavorable effects were demonstrated in the donepezil-treated OVX rats in relation to the OVX controls. The only statistically significant effects of donepezil on the OVX rats’ skeletal system demonstrated in the present study were increased phosphorus content in the bone mineral of the femur and increased width of the hypertrophic zone in the epiphyseal cartilage compared to the OVX control rats.

The interaction between bone and skeletal muscle is well recognized and many clinical studies have shown an association between osteoporosis and sarcopenia [41,76]. Since the prevalence of sarcopenia was reported to be higher in patients with AD than in patients with normal cognition [77] and a recent systematic review reported an increased prevalence of frailty and sarcopenia in patients with dementia (including AD) [78], we also examined the effect of donepezil on the skeletal muscle. Looking for a possible mechanism of donepezil action on the musculoskeletal system, we hypothesized that donepezil, by blocking AchE, would affect the neuromuscular transmission and parameters related to the muscular system. In adult mice, donepezil impaired neuromuscular function by generating prolonged aftercontractions after brief stimulation at moderate frequencies and by prolonging synaptic depolarizations associated with an increased incidence of spontaneous muscle contractions [79]. Donepezil has also been shown to exert adverse effects on muscles, manifested through muscle cramps in patients [80,81]. On the other hand, donepezil promoted muscle differentiation independent of its AchE-inhibitory action in C2C12 mouse myoblast cells in vitro and in a mouse model of a cardiotoxin-based skeletal muscle injury in vivo [82]. In the present study, donepezil tended to decrease the grip strength in NOVX rats compared to the NOVX controls. Such a result may suggest an unfavorable effect of donepezil on bones through the effects on the skeletal muscle strength in rats with normal estrogen levels. On the contrary, such an effect was not observed in estrogen-deficient rats. Our results obtained in NOVX rats, suggesting potential unfavorable effects of donepezil on the muscular system, are consistent with other clinical reports, where overdose and/or high doses of donepezil caused muscle weakness [83,84].

Interestingly, the present study demonstrated differential, estrogen-dependent effects of donepezil on the epiphyseal cartilage (growth plate). Donepezil exerted an adverse effect on the epiphyseal cartilage by reducing the width of the hypertrophic zone in rats with normal estrogen levels. In estrogen-deficient rats, the opposite effect was observed—donepezil increased the width of the hypertrophic zone of the epiphyseal cartilage, decreased due to estrogen deficiency. To our knowledge, this is the first observation concerning donepezil’s effects on the epiphyseal cartilage. Nicotine was reported to reduce hypertrophic differentiation of growth plate chondrocytes in vitro [85]. It has been reported that cholinergic system activation increased chondrocyte proliferation and delayed chondrocyte differentiation [86]. Donepezil was reported to exert a chondroprotective effect in osteoarthritis in vitro by suppressing TNF-α-induced expression of matrix metalloproteinase-13 [87]. However, articular cartilage differs in structure from epiphyseal cartilage (growth plate) and their roles are different. The growth plate is responsible for the longitudinal growth of the long bone, while the articular cartilage is responsible for the elasticity and smooth movement within the joints [88]. It should be also pointed out that the effect of donepezil on the epiphyseal cartilage in both NOVX and OVX rats may be irrelevant to the clinical conditions since the drug is used mostly in elderly patients and the rats used in the present study were still growing.

In order to clarify the mechanism of changes in the musculoskeletal system induced by donepezil, an analysis of the serum levels of cytokines was performed. Only the VEGF level increased in the OVX controls compared to the NOVX controls. Donepezil administration to NOVX rats did not affect cytokine levels. In the donepezil-treated OVX rats, the levels of chemokines CCL20 (MIP-3a) and CCL2 (MCP-1) increased compared to the OVX controls.

Chemokines are proinflammatory chemotactic cytokines [89]. CCL2 (also known as MCP-1) is a chemokine produced by vascular endothelial, epithelial, smooth muscle cells, fibroblastic, astrocytic, monocytic, microglial cells and osteoblasts [90]. The neuronal expression of CCL2 was found in the brain [91]. CCL2 is involved in the bone remodeling process (stimulating osteoclast differentiation and maturation) [90]. CCL20 (also known as MIP-3a) is widely expressed in peripheral blood mononuclear cells, neutrophils, eosinophils, mast cells, dendritic cells, macrophages, epithelial cells, keratinocytes, melanocytes, fibroblasts [92] and in bone cells [93]. CCL20 increased bone resorption and bone formation (through the stimulation of osteoclastogenesis and increased osteoblast differentiation) [93,94]. In summary, the results concerning the effects of donepezil on the serum levels of cytokines in OVX rats suggest a potential unfavorable effect on bones due to increased expression of proinflammatory cytokines. The results of the present study are inconsistent with the results of other studies, where donepezil demonstrated anti-inflammatory activity in vitro by reducing the expression of IL-1β, TNF-α mRNA and NF-κB signaling in LPS-stimulated microglial cells [95] and reduced MCP-1 (CCL2) and IL-4 mRNA expression in peripheral blood mononuclear cells from AD patients [96], whereas, consistently with our study, no effect of galantamine (BBB-permeable AchEI such as donepezil) was observed on the bone IL-1β, IL-6 or TNF-α mRNA expression in female mice [70].

The reasons for the differential effects of donepezil in our study and other studies [37,38,39,69] can be speculated. The doses used in the long-term experimental studies were very similar. Taking into account the oral bioavailability of donepezil in rats [97], the dose used in the present study seems to be equivalent to the dose of 0.6 mg/kg sc. used in another experimental study on the skeletal system in rats [37]. In an experimental study in mice, a donepezil dose of 0.6 mg/kg i.p. was used [38]; assuming higher bioavailability of donepezil after i.p. than p.o. administration and faster metabolism in mice [55], this dose was also close to that used in the present study. The similarity of doses used in mouse and rat studies indicates the possibility of species differences. In rats, a mechanism related to the stimulation of nicotinic receptors in the autonomic nervous system ganglia may be responsible for the effects of donepezil (a greater component resulting from sympathetic system activation than in mice). The complexity of the effect of the cholinergic system on the musculoskeletal system requires more studies. We speculate that the donepezil effects observed in rats may have resulted from the predominance of the sympathetic system over the parasympathetic system activation in NOVX rats.

A limitation of the present study is that it was performed on relatively young rats. Taking this into consideration, the study should be treated as preliminary and, based on its results, further studies may be planned.

In conclusion, although donepezil exerted a slight adverse effect on the musculoskeletal system in rats with normal estrogen levels, it showed no deleterious effect in conditions of estrogen deficiency. The results of the present study on rats may indicate the relative musculoskeletal safety of donepezil treatment in postmenopausal women with AD and other diseases.

## 4. Materials and Methods

The study was carried out on 40 sexually mature (15 weeks old at the start of the experiment) female Wistar rats bred in the Center of Experimental Medicine, Medical University of Silesia, Katowice, Poland.

All experimental procedures were approved by the Local Ethics Committee, Katowice, Poland (permission number: 30/2019). The study was carried out in the Center of Experimental Medicine, Medical University of Silesia, Katowice, Poland where the animals were provided with optimal living conditions, complying with the European Union guidelines (Directive 2010/63/EU), which were regulated and monitored every day (temperature in the range of 22–24 °C, relative air humidity—in the range from 50 to 60%, photoperiod cycle lights from 7:00 a.m. to 7:00 p.m.). The animals were housed five per standard plastic cage. The rats had ad libitum access to a standard laboratory diet (Labofeed B, Wytwórnia Pasz Morawski, Kcynia, Poland) and drinking (tap) water, as well as sanitary and hygienic services during the acclimatization period and throughout the experiment. The study shares control groups with our other study [98].

The rats were randomized after the acclimatization period, and before performing the bilateral ovariectomy, into four groups (10 rats per group):

I—non-ovariectomized control rats (NOVX group),

II—NOVX rats treated with donepezil (NOVX + D group),

III—ovariectomized control rats (OVX group),

IV—OVX rats treated with donepezil (OVX + D group).

The following drugs were used during the experiment: donepezil (Yasnal, coated tablets, 10 mg of donepezil hydrochloride (9.12 mg of donepezil), KRKA d. d., Nove Mesto, Slovenia); drugs used for general anesthesia: ketamine (Ketamina Biowet Puławy, injection solution, 100 mg/mL, Biowet Puławy Sp. z o.o., Puławy, Poland) and xylazine (Sedazin, injection solution, 20 mg/mL, Biowet Puławy Sp. z o.o., Puławy, Poland); and a drug used to mark the calcification front in bone: tetracycline hydrochloride (Sigma-Aldrich Co., St. Louis, MO, USA).

The bilateral ovariectomy was performed at the start of the experiment (after the acclimatization period) under general anesthesia (caused by intraperitoneal (i.p.) injection of ketamine and xylazine mixture) in rats from group III and IV. Drug administration started one week after the surgery and lasted four weeks. Donepezil was administered orally (p.o., by oral gavage) at a dose of 1 mg/kg, in a volume of 2 mL/kg as a suspension in tap water once daily in the morning hours to rats from group II and IV. Rats from group I and III were administered tap water (vehicle) in a volume of 2 mL/kg p.o. In order to mark the calcification front, tetracycline hydrochloride was administered at a dose of 20 mg/kg i.p. seven days after the ovariectomy (on the first day of drug or vehicle administration) and on the last day of drug or vehicle administration. Rats were weighed at the start of the experiment (no differences in the initial body mass between groups were noted), one week after the ovariectomy and then once a week on an analytical balance (type PS 2100.X2; Radwag, Radom, Poland). Body mass gain after 4 weeks of drug or vehicle administration was determined.

Moreover, to measure the skeletal muscle strength, the forelimb grip strength was measured on the last day of drug or vehicle administration with the use of a grip strength meter apparatus for rats and mice (model 47200; Ugo Basile, Gemonio, Italy). The rat, after grasping the T-shaped apparatus bar, was pulled by the tail by the experimenter until the pulling force overcame the strength of the grip. At this point, the apparatus automatically measured the peak force achieved by the forelimbs. Data were monitored, transferred and analyzed using a computer with data collection application (DCA) software version 2.2 (Ugo Basile, Gemonio, Italy), as previously described [57].

The experiment was terminated five weeks after the ovariectomy (after four weeks of drug or vehicle administration). The rats were fasted overnight with unrestricted access to drinking water and sacrificed under general anesthesia (rats were anesthetized with ketamine and xylazine mixture i.p. injection) by cardiac exsanguination. The obtained blood was centrifuged and the serum was frozen at a temperature of −80 °C to determine the biochemical parameters. During an autopsy, internal organs (liver, uterus and thymus), as well as bones (left and right femurs and tibias and L4 vertebras) and skeletal muscles (musculus soleus, musculus gastrocnemius, musculus tibialis anterior) were isolated and weighed on the Adventurer Pro type AV264CM analytical balance (Ohaus Europe GmbH, Greifensee, Switzerland). The length of the left tibia and left femur of each rat was determined using a digital caliper (Topex, Warsaw, Poland). The left tibia, left femur, L4 vertebra and a proximal part of the right femur of each rat were wrapped in cotton gauze soaked in saline and stored at a temperature of −20 °C for further studies. The right tibia and a distal part of the right femur of each rat were used for the determination of the histomorphometric parameters.

### 4.1. Biochemical Studies

The serum level of bone resorption marker—C-terminal telopeptide fragments of type I collagen (RatLaps CTX-I EIA) and the serum level of bone formation marker–osteocalcin (Rat-MID Osteocalcin EIA) were determined using ELISA kits (Immunodiagnostic Systems Ltd., Boldon, UK). A Tecan Sunrise microplate reader with Magellan v7.0 software (Tecan Austria GmbH, Grödig, Austria) was used for the measurements.

The serum concentrations of calcium, phosphorus, total cholesterol, total protein, uric acid, urea, glucose and the activity of ALP, lactate dehydrogenase (LDH), ALT and aspartate aminotransferase (AST) in the serum were measured using an automatic biochemical analyzer (Mindray BS-240, Shenzhen, China) with commercially available kits (Pointe Scientific, Canton, MI, USA); the serum level of creatinine was determined using a reagent produced by Thermo Fisher Scientific Inc., Middletown, WI, USA.

Bio-Plex 200 system and Bio-Plex Manager Software (Bio-Rad Laboratories Inc., Hercules, CA, USA) were used for the measurement of the levels of 23 rat cytokines in the serum by means of multiplex technology of magnetic bids combined with antibodies (Bio-Plex Pro Rat Cytokine 23-Plex Immunoassay; Bio-Rad Laboratories Inc., Hercules, CA, USA). The following cytokines were evaluated: interleukin-1α (IL-1α), IL-1β, IL-2, IL-4, IL-5, IL-6, IL-7, IL-10, IL-12p70, IL-13, IL-17A, IL-18, macrophage colony-stimulating factor (M-CSF), granulocyte colony-stimulating factor (G-CSF), granulocyte-macrophage colony-stimulating factor (GM-CSF), interferon-γ (IFN-γ), tumor necrosis factor α (TNF-α), vascular endothelial growth factor (VEGF), monocyte chemoattractant protein-1 (MCP-1; CC motif chemokine ligand 2 (CCL2)), macrophage inflammatory protein-1a (MIP-1a; CC motif chemokine ligand 3 (CCL3)), regulated on activation, normal T cell expressed and secreted (RANTES; CC motif chemokine ligand 5 (CCL5)), macrophage inflammatory protein-3a (MIP-3a; CC motif chemokine ligand 20 (CCL20)) and growth-regulated oncogene/keratinocyte chemoattractant (GRO/KC; CXC motif chemokine ligand 1 (CXCL1)).

### 4.2. Bone Mechanical Properties Studies

The mechanical properties studies were performed on the left tibia (the proximal tibial metaphysis), left femur (the femoral diaphysis) and the proximal part of the right femur (the femoral neck) of each rat using Instron 3342 500 N apparatus (Instron, Norwood, MA, USA). The data were analyzed using Bluehill 2 software version 2.14 (Instron, Norwood, MA, USA) [58,99,100,101].

Three-point bending tests were used to evaluate the mechanical properties of the left proximal tibial metaphysis (cancellous bone) and left femoral diaphysis (compact bone), while a compression test was used to determine the strength of the right femoral neck. The displacement rate for the tests was 0.01 mm/s and the sampling rate was 100 Hz.

The mechanical properties of the proximal tibial metaphysis were evaluated after removing the proximal tibial epiphysis, using a three-point bending test [58,99,100,101,102]. The two supporting points were the edge of the proximal tibial metaphysis and the location of the fibula attachment to the tibia. The distance between those points was measured using a digital caliper (Topex) before each test. The load was applied 3 mm from the edge of the proximal tibial metaphysis perpendicularly to the longitudinal bone axis. Before the proper test, a preload of 1 N was applied to stabilize the bone. To calculate the moment of inertia in the break-section, necessary for the calculation of intrinsic bone parameters, it was assumed that the cross-section of the proximal tibial metaphysis at the fracture site had a shape of a circular beam. The mean diameter of the tibial metaphysis (3 mm from the edge) was determined using a digital caliper. The following parameters were determined: the load, displacement, energy and stress at three points: the yield point (0.05% offset), the maximum load point and the fracture point. Young’s modulus was also determined.

To evaluate the mechanical properties of the femoral diaphysis, the load was applied in the middle of the bone perpendicularly to the longitudinal bone axis [58,99,100,101,103]. The distance between supporting points was 16 mm. A proper test was preceded by the application of a preload to stabilize the bone (five cycles of the preload from 0 to 4 N). To calculate the moment of inertia in the break-section, it was assumed that the femoral diaphysis was an elliptical pipe [104]. The measurements of internal and external diameters were made in the transverse cross-sections of the right femoral diaphysis (preparations were made in the middle of the bone length) using the OsteoMeasure system with OsteoMeasure XP v1.3.0.1 software (OsteoMetrics, Decatur, GA, USA). The same parameters as for the proximal tibial metaphysis were determined.

To evaluate the mechanical properties of the femoral neck, the bone was permanently attached to the polymethyl methacrylate plate. The load was applied to the head of the femur along the longitudinal bone axis and the maximum load (inducing fracture of the femoral neck) was determined [58,99,100,101].

### 4.3. Bone Composition and Mineralization Studies

After performing the mechanical properties studies, the bones (left tibia deprived of the proximal epiphysis, left femur and L4 vertebra) of each rat were lyophilized for ten days in FreeZone 6 lyophilizer (Labconco, Kansas City, MO, USA) at a pressure of 0.03 mBar and a temperature of −51 °C. To assess the mass of water in bone (calculated by subtracting the bone mass after lyophilization from the bone mass), the bone mass after lyophilization was determined using the Adventurer Pro type AV264CM analytical balance. After lyophilization, the bones were ashed at a temperature of 640 °C for 48 h in a L9/11/C6 muffle furnace (Nabertherm, Lilienthal, Germany) and weighed to determine the bone mineral mass. Bone organic substance mass was calculated by subtracting the bone mineral mass from the bone mass after lyophilization. The ratios of the bone mineral, water and organic substance to the bone mass were also calculated [57,99,100].

The ashed bones were dissolved in 6 M HCl and then diluted with deionized water to determine calcium, phosphorus and magnesium content in the bone mineral, using an automatic biochemical analyzer (Mindray BS-240, Shenzhen, China) with commercially available kits (for calcium and phosphorus: Pointe Scientific, Canton, MI, USA; for magnesium: Alpha Diagnostics Sp. z o.o., Warsaw, Poland). Calcium, phosphorus and magnesium contents were calculated based on the conversion of the obtained concentrations in the prepared dilutions.

Bone density was determined in the left tibia deprived of the proximal epiphysis and left femur (before the mechanical tests) and in the L4 vertebra using the Adventurer Pro type AV264CM analytical balance with a density determination kit (Ohaus Europe GmbH, Greifensee, Switzerland) based on the Archimedes’ principle [105]. Bone mineral density was calculated as the ratio of bone mineral mass to bone volume.

### 4.4. Bone Histomorphometric Studies

The histomorphometric studies were performed using an OsteoMeasure System, including an Axio Imager.A1 microscope (Carl Zeiss, Göttingen, Germany), Olympus DP71 camera (Olympus, Tokyo, Japan), Cintiq 22HD graphic tablet (Wacom, Kazo, Japan) and computer with OsteoMeasure XP v1.3.0.1 software (OsteoMetrics, Decatur, GA, USA). In addition, to measure the periosteal mineral apposition rate (Ps.MAR) under UV light, a set consisting of an Optiphot-2 microscope (Nikon, Japan), DS-Fi3 camera (Nikon, Tokyo, Japan), HB-10104 AF UV source (Nikon, Japan) and NIS-Elements D version 5.20.00 software (Nikon, Melville, NY, USA) was used. The histomorphometric parameters were presented according to the American Society for Bone and Mineral Research (ASBMR) standardized nomenclature [106].

The measurements of the transverse cross-sections of the tibial and femoral diaphysis were carried out on undecalcified, unstained preparations, prepared as previously described [58,99,100,107]. The following histomorphometric parameters were measured in the tibial and femoral diaphysis: the transverse cross-sectional area of the cortical bone (Ct.Ar), transverse cross-sectional area of the marrow cavity (Ma.Ar), transverse cross-sectional area of the total diaphysis (Tt.Ar) and the transverse cross-sectional area of the marrow cavity/total diaphysis area ratio (Ma.Ar/Tt.Ar). Moreover, the Ps.MAR in the femoral diaphysis was determined based on the measurements of the mean distance between the tetracycline labels incorporated into the bone divided by the number of days separating the administration of tetracycline hydrochloride.

The measurements of the longitudinal cross-sections of the distal femoral epiphysis and metaphysis and epiphyseal cartilage were performed on decalcified and stained with hematoxylin and eosin preparations, as previously described [57,99,100]. The following histomorphometric parameters were measured in cancellous bone (the distal femoral metaphysis and epiphysis): bone volume/tissue volume ratio (BV/TV), trabecular thickness (Tb.Th), trabecular separation (Tb.Sp) and trabecular number (Tb.N). In addition, the width of the reserve, proliferative and hypertrophic zones of the distal epiphyseal cartilage were determined.

### 4.5. Statistical Analysis

Results are presented as the mean ± standard error of the mean (SEM). Statistical analysis was carried out using two-way analysis of variance (ANOVA), with the main effects of estrogen deficiency (OVX) and donepezil treatment (D). In case of the statistical significance of any of the main effects or their interaction (OVXxD), the ANOVA was followed by Fisher’s Least Significant Difference (LSD) post hoc test (Statistica 13.3; Tibco Software Inc., Palo Alto, CA, USA). *p* values < 0.05 were considered to be statistically significant. *p* values < 0.1 in the LSD post hoc test were described as tendencies.

## Figures and Tables

**Table 1:** Effects of donepezil (1 mg/kg p.o.) administered for 4 weeks on the body mass gain and mass of internal organs in non-ovariectomized and ovariectomized rats.

Parameter/Group	NOVX	NOVX + D	OVX	OVX + D	Two-Way ANOVA		
					OVX	D	OVXxD
Initial body mass (g)	210.1 ± 5.2	204.7 ± 4.2	207.5 ± 2.8	207.4 ± 5.0	NS	NS	NS
Body mass gain after 4 weeks (g)	11.2 ± 2.2	7.0 ± 1.7	33.4 ± 4.8 ***	29.6 ± 3.2 ***	*p* < 0.001	NS	NS
Mass of liver (g)	5.42 ± 0.14	5.29 ± 0.13	5.52 ± 0.16	5.60 ± 0.09	NS	NS	NS
Mass of uterus (g)	0.606 ± 0.063	0.585 ± 0.073	0.083 ± 0.003 ***	0.081 ± 0.003 ***	*p* < 0.001	NS	NS
Mass of thymus (g)	0.233 ± 0.021	0.228 ± 0.009	0.383 ± 0.029 ***	0.341 ± 0.031 **	*p* < 0.001	NS	NS

The results are presented as means ± standard error of the mean (SEM). NOVX—non-ovariectomized control rats; NOVX + D—non-ovariectomized rats treated with donepezil; OVX—ovariectomized control rats; OVX + D—ovariectomized rats treated with donepezil. The body mass gain concerned the period of the drug treatment (to the day preceding the euthanasia). Two-way analysis of variance (ANOVA) followed by Fisher’s LSD test were used for evaluation of the significance of the results. NS—non-significant in the two-way ANOVA. ** *p* < 0.01, *** *p* < 0.001—in comparison to the NOVX control rats (NOVX group).

**Table 2:** Effects of donepezil (1 mg/kg p.o.) administered for 4 weeks on the serum general biochemical parameters in non-ovariectomized and ovariectomized rats.

Parameter/Group	NOVX	NOVX + D	OVX	OVX + D	Two-Way ANOVA		
					OVX	D	OVXxD
LDH (U/L)	1371 ± 138	958 ± 130	981 ± 136	1478 ± 213 ^#^	NS	NS	*p* < 0.01
ALT (U/L)	25.3 ± 3.7	29.6 ± 3.6	17.7 ± 0.9	22.9 ± 2.6	*p* < 0.05	NS	NS
AST (U/L)	151.9 ± 9.2	178.2 ± 10.9	162.9 ± 11.4	180.0 ± 14.0	NS	NS	NS
Fasting glucose (mg/dL)	72.7 ± 4.2	75.3 ± 4.8	73.8 ± 3.4	63.1 ± 4.1	NS	NS	NS
Total cholesterol (mg/dL)	51.2 ± 1.8	48.3 ± 4.5	61.3 ± 2.2 *	56.9 ± 3.6	*p* < 0.01	NS	NS
Total protein (g/dL)	7.36 ± 0.19	7.29 ± 0.26	6.95 ± 0.26	6.72 ± 0.20 *	*p* < 0.05	NS	NS
Uric acid (mg/dL)	2.00 ± 0.24	1.61 ± 0.24	1.37 ± 0.11 *	1.37 ± 0.12 *	*p* < 0.05	NS	NS
Urea (mg/dL)	32.1 ± 1.4	33.7 ± 1.1	30.1 ± 1.4	28.0 ± 1.5 *	*p* < 0.01	NS	NS
Creatinine (mg/dL)	0.55 ± 0.04	0.55 ± 0.04	0.51 ± 0.03	0.45 ± 0.03	NS	NS	NS

The results are presented as means ± standard error of the mean (SEM). NOVX—non-ovariectomized control rats; NOVX + D—non-ovariectomized rats treated with donepezil; OVX—ovariectomized control rats; OVX + D—ovariectomized rats treated with donepezil. LDH—lactate dehydrogenase; ALT—alanine aminotransferase; AST—aspartate aminotransferase. Two-way analysis of variance (ANOVA) followed by Fisher’s LSD test were used for evaluation of the significance of the results. NS—non-significant in the two-way ANOVA. * *p* < 0.05—in comparison to the NOVX control rats (NOVX group). ^#^ *p* < 0.05—in comparison to the OVX control rats (OVX group).

**Table 3:** Effects of donepezil (1 mg/kg p.o.) administered for 4 weeks on the serum bone metabolic parameters in non-ovariectomized and ovariectomized rats.

Parameter/Group	NOVX	NOVX + D	OVX	OVX + D	Two-Way ANOVA		
					OVX	D	OVXxD
Osteocalcin (ng/mL)	257.9 ± 18.8	264.4 ± 18.5	372.4 ± 12.9 ***	390.7 ± 26.2 ***	*p* < 0.001	NS	NS
CTX-I (ng/mL)	15.06 ± 1.50	15.84 ± 1.26	23.71 ± 2.09 **	25.06 ± 2.72 **	*p* < 0.001	NS	NS
ALP (U/L)	65.3 ± 6.4	60.4 ± 5.6	97.2 ± 6.2 ***	89.4 ± 4.2 **	*p* < 0.001	NS	NS
Ca (mg/dL)	10.37 ± 0.31	10.77 ± 0.39	10.53 ± 0.26	10.26 ± 0.30	NS	NS	NS
P (mg/dL)	4.76 ± 0.34	6.58 ± 0.20 ***	6.85 ± 0.18 ***	6.76 ± 0.19 ***	*p* < 0.001	*p* < 0.001	*p* < 0.001

The results are presented as means ± standard error of the mean (SEM). NOVX—non-ovariectomized control rats; NOVX + D—non-ovariectomized rats treated with donepezil; OVX—ovariectomized control rats; OVX + D—ovariectomized rats treated with donepezil. CTX-I—C-terminal telopeptide fragments of type I collagen; ALP—alkaline phosphatase; Ca—calcium; P—phosphorus. Two-way analysis of variance (ANOVA) followed by Fisher’s LSD test were used for evaluation of the significance of the results. NS—non-significant in the two-way ANOVA. ** *p* < 0.01, *** *p* < 0.001—in comparison to the NOVX control rats (NOVX group).

**Table 4:** Effects of donepezil (1 mg/kg p.o.) administered for 4 weeks on mechanical properties of the femoral diaphysis and femoral neck in non-ovariectomized and ovariectomized rats.

Parameter/Group		NOVX	NOVX + D	OVX	OVX + D	Two-Way ANOVA		
						OVX	D	OVXxD
Femoral diaphysis	Yield point load (N)	84.2 ± 3.6	81.5 ± 2.2	80.9 ± 3.0	79.0 ± 3.6	NS	NS	NS
	Displacement for yield point load (mm)	0.305 ± 0.016	0.292 ± 0.018	0.279 ± 0.010	0.272 ± 0.012	NS	NS	NS
	Energy for yield point load (mJ)	11.7 ± 0.8	11.2 ± 0.7	10.6 ± 0.6	10.3 ± 0.6	NS	NS	NS
	Stress for yield point load (MPa)	127.5 ± 5.6	130.5 ± 5.4	127.4 ± 5.0	115.6 ± 5.0	NS	NS	NS
	Maximum load (N)	106.9 ± 3.7	109.3 ± 2.6	108.4 ± 5.5	113.0 ± 4.5	NS	NS	NS
	Displacement for maximum load (mm)	0.457 ± 0.024	0.491 ± 0.033	0.454 ± 0.030	0.509 ± 0.015	NS	NS	NS
	Energy for maximum load (mJ)	26.2 ± 1.8	30.7 ± 3.3	27.8 ± 3.3	33.6 ± 2.6	NS	NS	NS
	Stress for maximum load (MPa)	161.7 ± 5.3	174.7 ± 6.2	170.2 ± 7.7	165.2 ± 5.4	NS	NS	NS
	Fracture load (N)	106.7 ± 3.7	108.6 ± 2.3	107.6 ± 4.9	112.7 ± 4.5	NS	NS	NS
	Displacement for fracture load (mm)	0.459 ± 0.024	0.514 ± 0.051	0.459 ± 0.034	0.514 ± 0.016	NS	NS	NS
	Energy for fracture load(mJ)	26.4 ± 1.8	33.5 ± 5.9	28.5 ± 3.8	34.3 ± 2.7	NS	NS	NS
	Stress for fracture load (MPa)	161.5 ± 5.2	173.7 ± 6.2	169.1 ± 7.4	164.8 ± 5.5	NS	NS	NS
Maximum load in the femoral neck (N)		69.5 ± 3.8	72.3 ± 4.1	70.1 ± 2.9	70.6 ± 3.3	NS	NS	NS

The results are presented as means ± standard error of the mean (SEM). NOVX—non-ovariectomized control rats; NOVX + D—non-ovariectomized rats treated with donepezil; OVX—ovariectomized control rats; OVX + D—ovariectomized rats treated with donepezil. Two-way analysis of variance (ANOVA) was used for evaluation of the significance of the results. NS—non-significant in the two-way ANOVA.

**Table 5:** Effects of donepezil (1 mg/kg p.o.) administered for 4 weeks on bone length, mass, composition, mineralization and density in the femur in non-ovariectomized and ovariectomized rats.

Parameter/Group	NOVX	NOVX + D	OVX	OVX + D	Two-Way ANOVA		
					OVX	D	OVXxD
Bone length (mm)	32.86 ± 0.21	32.64 ± 0.20	32.90 ± 0.34	33.06 ± 0.20	NS	NS	NS
Bone mass (g)	0.647 ± 0.013	0.646 ± 0.008	0.634 ± 0.019	0.638 ± 0.013	NS	NS	NS
Bone mineral mass (g)	0.300 ± 0.006	0.298 ± 0.005	0.282 ± 0.008	0.281 ± 0.009	*p* < 0.05	NS	NS
Bone mineral mass/ bone mass ratio	0.465 ± 0.003	0.461 ± 0.005	0.446 ± 0.004 *	0.439 ± 0.008 **	*p* < 0.001	NS	NS
Mass of bone water/ bone mass ratio	0.304 ± 0.004	0.311 ± 0.008	0.326 ± 0.004	0.336 ± 0.013 **	*p* < 0.01	NS	NS
Mass of bone organic substance/bone mass ratio	0.231 ± 0.002	0.228 ± 0.003	0.228 ± 0.002	0.225 ± 0.005	NS	NS	NS
Calcium content (g/g of bone mineral)	0.425 ± 0.003	0.428 ± 0.003	0.428 ± 0.004	0.429 ± 0.003	NS	NS	NS
Phosphorus content (g/g of bone mineral)	0.172 ± 0.001	0.170 ± 0.001	0.170 ± 0.001	0.174 ± 0.002 ^#^	NS	NS	*p* < 0.01
Magnesium content (g/g of bone mineral)	0.011 ± 0.001	0.011 ± 0.001	0.011 ± 0.001	0.012 ± 0.001	NS	NS	NS
Bone density (g/cm^3^)	1.618 ± 0.018	1.591 ± 0.009	1.553 ± 0.008 ***	1.548 ± 0.014 ***	*p* < 0.001	NS	NS
Bone mineral density (g/cm^3^)	0.729 ± 0.010	0.714 ± 0.008	0.662 ± 0.009 ***	0.662 ± 0.012 ***	*p* < 0.001	NS	NS

The results are presented as means ± standard error of the mean (SEM). NOVX—non-ovariectomized control rats; NOVX + D—non-ovariectomized rats treated with donepezil; OVX—ovariectomized control rats; OVX + D—ovariectomized rats treated with donepezil. Two-way analysis of variance (ANOVA) followed by Fisher’s LSD test were used for evaluation of the significance of the results. NS—non-significant in the two-way ANOVA. * *p* < 0.05, ** *p* < 0.01, *** *p* < 0.001—in comparison to the NOVX control rats (NOVX group). ^#^ *p* < 0.05—in comparison to the OVX control rats (OVX group).

**Table 6:** Effects of donepezil (1 mg/kg p.o.) administered for 4 weeks on histomorphometric parameters of compact bone in the femoral diaphysis in non-ovariectomized and ovariectomized rats.

Parameter/Group	NOVX	NOVX + D	OVX	OVX + D	Two-Way ANOVA		
					OVX	D	OVXxD
Ct.Ar (mm^2^)	5.001 ± 0.076	4.812 ± 0.092	4.845 ± 0.118	4.944 ± 0.103	NS	NS	NS
Ma.Ar (mm^2^)	2.532 ± 0.098	2.633 ± 0.065	2.782 ± 0.109	2.895 ± 0.114 *	*p* < 0.05	NS	NS
Tt.Ar (mm^2^)	7.533 ± 0.135	7.445 ± 0.097	7.627 ± 0.195	7.839 ± 0.111	NS	NS	NS
Ma.Ar/Tt.Ar	0.335 ± 0.009	0.354 ± 0.008	0.364 ± 0.009 *	0.369 ± 0.012 *	*p* < 0.05	NS	NS
Ps.MAR (μm/day)	1.37 ± 0.07	1.47 ± 0.10	1.57 ± 0.07	1.61 ± 0.09	*p* < 0.05	NS	NS

The results are presented as means ± standard error of the mean (SEM). NOVX—non-ovariectomized control rats; NOVX + D—non-ovariectomized rats treated with donepezil; OVX—ovariectomized control rats; OVX + D—ovariectomized rats treated with donepezil. Ct.Ar—transverse cross-sectional area of the cortical bone; Ma.Ar—transverse cross-sectional area of the marrow cavity; Tt.Ar—transverse cross-sectional area of the total diaphysis; Ma.Ar/Tt.Ar—transverse cross-sectional area of the marrow cavity/total diaphysis area ratio; Ps.MAR—periosteal mineral apposition rate. Two-way analysis of variance (ANOVA) followed by Fisher’s LSD test were used for evaluation of the significance of the results. NS—non-significant in the two-way ANOVA. * *p* < 0.05—in comparison to the NOVX control rats (NOVX group).

**Table 7:** Effects of donepezil (1 mg/kg p.o.) administered for 4 weeks on the width of the epiphyseal cartilage zones in the femur in non-ovariectomized and ovariectomized rats.

Parameter/Group	NOVX	NOVX + D	OVX	OVX + D	Two-Way ANOVA		
					OVX	D	OVXxD
Reserve zone (μm)	30.65 ± 1.48	30.30 ± 1.66	28.92 ± 1.06	31.69 ± 2.00	NS	NS	NS
Proliferative zone (μm)	22.98 ± 1.17	22.55 ± 1.00	25.63 ± 1.41	24.86 ± 1.36	NS	NS	NS
Hypertrophic zone (μm)	26.96 ± 0.80	23.40 ± 0.86 *	22.58 ± 1.01 *	28.33 ± 1.91 ^##^	NS	NS	*p* < 0.001

The results are presented as means ± standard error of the mean (SEM). NOVX—non-ovariectomized control rats; NOVX + D—non-ovariectomized rats treated with donepezil; OVX—ovariectomized control rats; OVX + D—ovariectomized rats treated with donepezil. Two-way analysis of variance (ANOVA) followed by Fisher’s LSD test were used for evaluation of the significance of the results. NS—non-significant in the two-way ANOVA. * *p* < 0.05—in comparison to the NOVX control rats (NOVX group). ^##^ *p* < 0.01—in comparison to the OVX control rats (OVX group).

**Table 8:** Effects of donepezil (1 mg/kg p.o.) administered for 4 weeks on the serum concentrations of cytokines in non-ovariectomized and ovariectomized rats.

Parameter/Group	NOVX	NOVX + D	OVX	OVX + D	Two-Way ANOVA		
					OVX	D	OVXxD
IL-1α (pg/mL)	95.5 ± 13.7	99.3 ± 12.6	97.8 ± 8.8	105.7 ± 8.7	NS	NS	NS
IL-1β (pg/mL)	196.9 ± 76.5	250.7 ± 84.6	448.2 ± 75.8	815.7 ± 248.6 **	*p* < 0.01	NS	NS
IL-2 (pg/mL)	64.6 ± 25.3	87.5 ± 30.4	68.5 ± 32.0	33.0 ± 17.4	NS	NS	NS
IL-4 (pg/mL)	59.3 ± 9.6	53.6 ± 10.5	47.5 ± 9.1	48.2 ± 8.3	NS	NS	NS
IL-5 (pg/mL)	289.8 ± 22.2	331.1 ± 34.8	304.1 ± 19.7	253.6 ± 36.9	NS	NS	NS
IL-6 (pg/mL)	4.93 ± 3.83	1.18 ± 0.15	1.09 ± 0.10	1.24 ± 0.30	NS	NS	NS
IL-7 (pg/mL)	439.4 ± 157.9	660.6 ± 267.9	1123.5 ± 195.1	1679.7 ± 368.2 **	*p* < 0.01	NS	NS
IL-10 (pg/mL)	34.6 ± 5.9	45.8 ± 8.1	50.8 ± 8.2	37.6 ± 4.9	NS	NS	NS
IL-12p70 (pg/mL)	58.9 ± 11.9	75.9 ± 12.0	62.9 ± 5.3	50.2 ± 7.6	NS	NS	NS
IL-13 (pg/mL)	4.11 ± 0.35	4.33 ± 0.47	4.25 ± 0.24	3.76 ± 0.22	NS	NS	NS
IL-17A (pg/mL)	7.27 ± 3.60	4.25 ± 0.85	10.99 ± 4.98	4.05 ± 2.32	NS	NS	NS
IL-18 (pg/mL)	175.3 ± 102.1	276.0 ± 115.9	249.3 ± 125.2	373.5 ± 179.7	NS	NS	NS
M-CSF (pg/mL)	0.98 ± 0.76	0.58 ± 0.25	0.19 ± 0.04	0.38 ± 0.10	NS	NS	NS
G-CSF (pg/mL)	0.11 ± 0.06	0.19 ± 0.10	0.08 ± 0.04	0.09 ± 0.06	NS	NS	NS
GM-CSF (pg/mL)	355.4 ± 120.2	520.5 ± 203.8	930.6 ± 166.6	1991.6 ± 787.8 *	*p* < 0.05	NS	NS
IFN-γ (pg/mL)	17.11 ± 6.14	21.88 ± 6.87	5.52 ± 3.24	7.13 ± 2.65	*p* < 0.05	NS	NS
TNF-α (pg/mL)	11.15 ± 5.06	6.06 ± 1.64	6.57 ± 3.29	25.88 ± 14.57	NS	NS	NS
VEGF (pg/mL)	190.1 ± 35.9	490.7 ± 159.4	965.7 ± 273.8 *	1028.7 ± 238.1 **	*p* < 0.01	NS	NS
MCP-1 (CCL2) (pg/mL)	2112 ± 699	12017 ± 8534	22559 ± 10419	67255 ± 24871 **^#^	*p* < 0.05	NS	NS
MIP-1a (CCL3) (pg/mL)	174.3 ± 69.5	1815.5 ± 1626.2	673.1 ± 199.6	2959.3 ± 1450.6	NS	NS	NS
RANTES (CCL5) (pg/mL)	416.9 ± 41.5	381.3 ± 31.2	444.6 ± 39.6	566.4 ± 110.1	NS	NS	NS
MIP-3a (CCL20) (pg/mL)	9.11 ± 1.48	9.75 ± 0.91	6.34 ± 0.61	10.62 ± 0.89 ^##^	NS	*p* < 0.05	NS
GRO/KC (CXCL1) (pg/mL)	375.0 ± 112.5	590.7 ± 206.3	877.8 ± 168.9	1451.2 ± 492.5 *	*p* < 0.05	NS	NS

The results are presented as means ± standard error of the mean (SEM). NOVX—non-ovariectomized control rats; NOVX + D—non-ovariectomized rats treated with donepezil; OVX—ovariectomized control rats; OVX + D—ovariectomized rats treated with donepezil. IL-1 α—interleukin-1α; IL-1β— interleukin-1β; IL-2—interleukin-2; IL-4—interleukin-4; IL-5—interleukin-5; IL-6—interleukin-6; IL-7—interleukin-7; IL-10—interleukin-10; IL-12p70—interleukin-12p70; IL-13—interleukin-13; IL-17A—interleukin-17A; IL-18—interleukin-18; M-CSF—macrophage colony-stimulating factor; G-CSF—granulocyte colony-stimulating factor; GM-CSF—granulocyte-macrophage colony-stimulating factor; IFN-γ—interferon-γ; TNF-α—tumor necrosis factor α; VEGF—vascular endothelial growth factor; MCP-1 (CCL2)—monocyte chemoattractant protein-1 (CC motif chemokine ligand 2); MIP-1a (CCL3)—macrophage inflammatory protein-1a (CC motif chemokine ligand 3); RANTES (CCL5)—regulated on activation, normal T cell expressed and secreted (CC motif chemokine ligand 5); MIP-3a (CCL20)—macrophage inflammatory protein-3a (CC motif chemokine ligand 20); GRO/KC (CXCL1)—growth-regulated oncogene/keratinocyte chemoattractant (CXC motif chemokine ligand 1). Two-way analysis of variance (ANOVA) followed by Fisher’s LSD test were used for evaluation of the significance of the results. NS—non-significant in the two-way ANOVA. * *p* < 0.05, ** *p* < 0.01—in comparison to the NOVX control rats (NOVX group). ^#^ *p* < 0.05, ^##^ *p* < 0.01—in comparison to the OVX control rats (OVX group).

## Data Availability

Data are contained within the article.

## Supplementary Materials

The following are available online at https://www.mdpi.com/article/10.3390/ijms24108991/s1.
