# Supplementary material for: Effects of Donepezil on the Musculoskeletal System in Female Rats
Source: Int J Mol Sci. 2023 May 19;24(10):8991. doi: 10.3390/ijms24108991 (PMC10218892; doi:10.3390/ijms24108991)
Supplement: Supplementary file 1 [file ijms-24-08991-s001.zip › ijms-2360268-supplementary.pdf]

Supplementary Table S1. Effects of donepezil (1 mg/kg p.o.) administered for 4 weeks on bone length, mass, composition, mineralization and density in the tibia deprived of the proximal epiphysis in non-ovariectomized and ovariectomized rats.

Parameter/Group	NOVX	NOVX + D	OVX	OVX + D	Two-Way ANOVA		
					OVX	D	OVXxD
Bone length [&] (mm)	36.04 ± 0.25	34.95 ± 0.76	36.55 ± 0.29	36.50 ± 0.16	$p < 0.05$	NS	NS
Bone mass (g)	0.396 ± 0.008	0.381 ± 0.009	0.389 ± 0.015	0.393 ± 0.005	NS	NS	NS
Bone mineral mass (g)	0.197 ± 0.004	0.195 ± 0.003	0.190 ± 0.005	0.192 ± 0.003	NS	NS	NS
Bone mineral mass/ bone mass ratio	0.497 ± 0.004	0.515 ± 0.017	0.491 ± 0.012	0.489 ± 0.005	NS	NS	NS
Mass of bone water/ bone mass ratio	0.256 ± 0.006	0.237 ± 0.027	0.262 ± 0.017	0.268 ± 0.009	NS	NS	NS
Mass of bone organic substance/bone mass ratio	0.247 ± 0.003	0.249 ± 0.010	0.247 ± 0.006	0.244 ± 0.005	NS	NS	NS
Calcium content (g/g of bone mineral)	0.414 ± 0.004	0.419 ± 0.002	0.415 ± 0.003	0.411 ± 0.007	NS	NS	NS
Phosphorus content (g/g of bone mineral)	0.168 ± 0.001	0.175 ± 0.007	0.165 ± 0.002	0.175 ± 0.009	NS	NS	NS
Magnesium content (g/g of bone mineral)	0.013 ± 0.001	0.012 ± 0.001	0.014 ± 0.001	0.012 ± 0.001	NS	NS	NS
Bone density (g/cm^3)	1.655 ± 0.020	1.653 ± 0.011	1.604 ± 0.012 *	1.606 ± 0.012 *	$p < 0.01$	NS	NS
Bone mineral density (g/cm^3)	0.805 ± 0.018	0.804 ± 0.009	0.751 ± 0.010 **	0.754 ± 0.009 **	$p < 0.001$	NS	NS

The results are presented as means ± standard error of the mean (SEM). [&]—the length of the whole bone. NOVX—non-ovariectomized control rats; NOVX + D—non-ovariectomized rats treated with donepezil; OVX—ovariectomized control rats; OVX + D—ovariectomized rats treated with donepezil. Two-way analysis of variance (ANOVA) followed by Fisher's LSD test were used for evaluation of the significance of the results. NS—non-significant in the two-way ANOVA. * $p < 0.05$, ** $p < 0.01$—in comparison to the NOVX control rats (NOVX group).

Supplementary Table S2. Effects of donepezil (1 mg/kg p.o.) administered for 4 weeks on bone mass, composition, mineralization and density in the L4 vertebra in non-ovariectomized and ovariectomized rats.

Parameter/Group	NOVX	NOVX + D	OVX	OVX + D	Two-Way ANOVA		
					OVX	D	OVXxD
Bone mass (g)	0.194 ± 0.009	0.195 ± 0.007	0.186 ± 0.007	0.188 ± 0.005	NS	NS	NS
Bone mineral mass (g)	0.076 ± 0.002	0.076 ± 0.002	0.070 ± 0.003	0.071 ± 0.002	$p < 0.05$	NS	NS
Bone mineral mass/ bone mass ratio	0.394 ± 0.014	0.392 ± 0.011	0.380 ± 0.011	0.379 ± 0.010	NS	NS	NS
Mass of bone water/ bone mass ratio	0.377 ± 0.021	0.378 ± 0.016	0.394 ± 0.018	0.391 ± 0.015	NS	NS	NS
Mass of bone organic substance/bone mass ratio	0.229 ± 0.008	0.230 ± 0.006	0.227 ± 0.007	0.230 ± 0.005	NS	NS	NS
Calcium content (g/g of bone mineral)	0.425 ± 0.003	0.416 ± 0.003 *	0.424 ± 0.003	0.419 ± 0.002	NS	$p < 0.05$	NS
Phosphorus content (g/g of bone mineral)	0.169 ± 0.002	0.167 ± 0.001	0.169 ± 0.001	0.169 ± 0.001	NS	NS	NS
Magnesium content (g/g of bone mineral)	0.010 ± 0.001	0.011 ± 0.000	0.011 ± 0.000	0.010 ± 0.001	NS	NS	NS
Bone density (g/cm^3)	1.434 ± 0.032	1.445 ± 0.026	1.399 ± 0.023	1.365 ± 0.019	$p < 0.05$	NS	NS
Bone mineral density (g/cm^3)	0.570 ± 0.018	0.542 ± 0.023	0.511 ± 0.025	0.497 ± 0.018 *	$p < 0.05$	NS	NS

The results are presented as means ± standard error of the mean (SEM). NOVX—non-ovariectomized control rats; NOVX + D—non-ovariectomized rats treated with donepezil; OVX—ovariectomized control rats; OVX + D—ovariectomized rats treated with donepezil. Two-way analysis of variance (ANOVA) followed by Fisher's LSD test were used for evaluation of the significance of the results. NS—non-significant in the two-way ANOVA. * $p < 0.05$—in comparison to the NOVX control rats (NOVX group).

Supplementary Table S3. Effects of donepezil (1 mg/kg p.o.) administered for 4 weeks on histomorphometric parameters of compact bone in the tibial diaphysis in non-ovariectomized and ovariectomized rats.

Parameter/Group	NOVX	NOVX + D	OVX	OVX + D	Two-Way ANOVA		
					OVX	D	OVXxD
Ct.Ar (mm^2)	3.260 ± 0.065	3.308 ± 0.063	3.416 ± 0.084	3.429 ± 0.048	$p < 0.05$	NS	NS
Ma.Ar (mm^2)	0.717 ± 0.042	0.704 ± 0.012	0.779 ± 0.038	0.827 ± 0.053	$p < 0.05$	NS	NS
Tt.Ar (mm^2)	3.977 ± 0.079	4.012 ± 0.063	4.195 ± 0.114	4.256 ± 0.067 *	$p < 0.01$	NS	NS
Ma.Ar/Tt.Ar	0.180 ± 0.009	0.176 ± 0.004	0.185 ± 0.005	0.194 ± 0.010	NS	NS	NS

The results are presented as means ± standard error of the mean (SEM). NOVX—non-ovariectomized control rats; NOVX + D—non-ovariectomized rats treated with donepezil; OVX—ovariectomized control rats; OVX + D—ovariectomized rats treated with donepezil. Ct.Ar—transverse cross-sectional area of the cortical area; Ma.Ar—transverse cross-sectional area of the marrow cavity; Tt.Ar—transverse cross-sectional area of the total diaphysis; Ma.Ar/Tt.Ar—transverse cross-sectional area of the marrow cavity/total diaphysis area ratio. Two-way analysis of variance (ANOVA) followed by Fisher's LSD test were used for evaluation of the significance of the results. NS—non-significant in the two-way ANOVA. * $p < 0.05$—in comparison to the NOVX control rats (NOVX group).
